# Biomarkers of oxidative stress and inflammation in urine samples of extremely preterm newborns and their association with risk for autism at age 6 months

**DOI:** 10.1192/j.eurpsy.2023.1222

**Published:** 2023-07-19

**Authors:** T. Bollain Muñoz, J. Merchán-Naranjo, B. Almansa, C. Chafer, M. Bento, D. Blanco-Bravo, A. Garcia-Blanco, L. Pina-Camacho

**Affiliations:** 1 Department of Child and Adolescent Psychiatry, Institute of Psychiatry and Mental Health, Hospital General Universitario Gregorio Marañon, CIBERSAM, IiSGM, School of Medicine, Universidad Complutense, Madrid; 2 Neonatal Research Group, Health Research Institute La Fe, University and Polytechnic Hospital La Fe, Valencia; 3 Neonatology Service, Hospital General Universitario Gregorio Marañon, IiSGM, School of Medicine, Universidad Complutense, Madrid, Spain

## Abstract

**Introduction:**

Extremely preterm birth (defined as birth before 28 weeks’ gestational age) has been associated with risk of developing autism spectrum disorder (ASD) in infancy. The underlying physiopathological pathways that condition the emergence of ASD on these kids remains unknown, although there is increasing evidence that oxidative stress and inflammation play an important role.

**Objectives:**

We investigated the association and the predictive value of marker levels with the primary outcome (risk for ASD at age 6 months, defined as presence of two or more clinical ASD “red flags” at this age), and with other demographic and clinical variables.

**Methods:**

In a sample of N= 68 extremely preterm newborns, we collected urine samples from birth up to first week of life (T1= birth, T2=24-72 hours, T3=day 7), and analysed levels of biomarkers of oxidative stress and inflammation, and assessed risk for ASD at age 6-months. Through liquid chromatography mass spectrometry, we obtained levels of lipid peroxidation, DNA and protein oxidation metabolites, alongside levels of inflammation markers.

**Results:**

Compared to those with no risk for ASD, patients at risk for ASD showed significantly higher levels of 14(RS)-14-F_4t_-NeuroP at 24-72 hours of life (d=1.296, p=.018) and significantly lower levels of total isoprostanes at 24 hours of life (d=1.161, p=.048). In patients at risk for ASD, levels of 14(RS)-14-F_4t_-NeuroP decreased significantly over time from 24-72 hours (T2) to day 7 of life (T3), p=.032.

**Conclusions:**

In summary, we obtained a panel of urine biomarkers potentially predictive of early risk for ASD in extremely preterm newborns.

**Disclosure of Interest:**

None Declared

